# Supplementary material for: Operator Performance of the Digital Setup Fabrication for Orthodontic–Orthognathic Treatment: An Explorative Study
Source: J Clin Med. 2021 Dec 28;11(1):145. doi: 10.3390/jcm11010145 (PMC8745578; doi:10.3390/jcm11010145)
Supplement: Supplementary file 1 [file jcm-11-00145-s001.zip › jcm-1512054-suppl.pdf]

Journal of
Clinical Medicine

Supplementary Material

Influence of extraction and SARME therapy on the outcome.

Tables S1 and S2 show data representing the influence of premolar extraction and SARME therapy on the reproducibility of the orthodontic setups.

Table S1. Influence of extraction therapy on the fabrication of the setup. Mean differences >0 mm indicate larger standard errors in extraction cases compared with non-extraction cases. Parameters roll, pitch and yaw in degrees and x,y,z in mm.

Jaw	Tooth type	Parameter	Mean of differences in standard error	95% CI		*p* value
				Lower	Upper	
Mandible	Incisors	roll	0.14	-0.70	0.98	0.632
		pitch	1.09	-0.65	2.84	0.141
		Yaw	2.31	-1.09	5.72	0.119
		X	0.36	-0.35	1.07	0.204
		Y	0.42	0.22	0.61	0.006
		Z	0.23	-0.29	0.76	0.247
	Canines	roll	1.41	-1.99	4.81	0.279
		pitch	0.54	-1.79	2.87	0.514
		Yaw	1.63	-0.01	3.27	0.051
		X	0.32	-0.15	0.79	0.118
		Y	0.66	0.39	0.93	0.004
		Z	0.12	-0.16	0.40	0.265
	Premolars	roll	0.46	-1.24	2.16	0.453
		pitch	0.06	-1.57	1.70	0.914
		Yaw	2.78	-0.19	5.76	0.059
		X	0.47	0.19	0.75	0.013
		Y	0.84	0.03	1.65	0.046
		Z	0.01	0.21	0.23	0.878
	Molars	roll	1.24	-0.08	2.57	0.058
		pitch	-1.60	-3.61	0.40	0.084
		Yaw	-0.14	-0.84	0.56	0.571
		X	0.50	0.16	0.83	0.018
		Y	0.81	-0.40	2.01	0.123
		Z	0.06	-0.41	0.52	0.714
Maxilla	Incisors	roll	0.45	-0.68	0.77	0.855
		pitch	0.18	-1.20	1.56	0.700
		Yaw	0.13	-1.76	2.03	0.836
		X	0.16	-0.14	0.46	0.187
		Y	0.29	-0.01	0.58	0.052
		Z	-0.08	-0.31	0.15	0.345
	Canines	roll	0.63	-1.40	2.66	0.395
		pitch	1.26	0.16	2.36	0.136
		Yaw	0.36	-1.87	2.59	0.642
		X	0.07	-0.32	0.46	0.620
		Y	0.35	0.07	0.63	0.028
		Z	-0.09	-0.31	0.13	0.285
	Premolars	roll	0.66	-0.67	1.99	0.212
		pitch	0.78	-1.28	1.43	0.867
		Yaw	0.62	-1.17	2.42	0.348

		X	0.29	-0.04	0.63	0.070
		Y	0.38	0.22	0.54	0.004
		Z	0.77	0.014	0.30	0.347
	Molars	roll	-0.13	-1.67	1.40	0.799
		pitch	-0.70	-2.77	1.36	0.358
		Yaw	0.50	-0.84	1.84	0.320
		X	0.42	-0.25	1.10	0.141
		Y	0.55	-0.22	1.32	0.106
		Z	0.03	-1.70	1.41	0.783

X left/right translation, Y anterior tot posterior translation, Z cranial/caudal ,CI confidence interval, Significance was set at $\alpha = 0.05$. Significant differences indicated in red.

Table S2. Influence of SARME therapy on the fabrication of the setup. Mean differences >0 mm or 0 degrees indicate larger standard errors in SARME cases compared with non-SARME cases. Parameters roll, pitch and yaw in degrees and x,y,z in mm.

Jaw	Tooth type	Parameter	Mean of differences in standard error	95% CI		*p* value
				Lower	Upper	
Mandible	Incisors	roll	-0.14	-1.70	1.41	0.783
Mandible		pitch	-1.11	-3.52	1.29	0.237
Mandible		Yaw	0.23	-4.82	5.27	0.895
Mandible		X	-0.18	-0.79	0.42	0.406
Mandible		Y	0.10	-0.65	0.85	0.696
Mandible		Z	0.03	-0.13	0.20	0.555
Mandible	Canines	roll	-0.23	-3.50	3.03	0.836
Mandible		pitch	-0.55	-3.31	2.21	0.568
Mandible		Yaw	0.37	-3.52	4.26	0.781
Mandible		X	-0.12	-0.83	0.58	0.613
Mandible		Y	0.02	-0.62	0.66	0.935
Mandible		Z	0.07	-0.19	0.32	0.468
Mandible	Premolars	roll	0.04	-1.73	1.81	0.948
Mandible		pitch	0.41	-0.93	1.75	0.404
Mandible		Yaw	-0.10	-4.64	4.44	0.950
Mandible		X	0.03	-0.42	0.47	0.857
Mandible		Y	0.13	-0.16	0.42	0.241
Mandible		Z	0.04	-0.15	0.22	0.586
Mandible	Molars	roll	1.97	0.37	3.57	0.030
Mandible		pitch	0.54	-1.71	2.78	0.501
Mandible		Yaw	0.74	0.23	1.25	0.019
Mandible		X	0.15	-0.16	0.46	0.220
Mandible		Y	0.26	-0.67	1.19	0.439
Mandible		Z	0.25	0.01	0.40	0.045
Maxilla	Incisors	roll	0.54	-0.40	1.48	0.165
Maxilla		pitch	0.69	-0.22	1.60	0.095
Maxilla		Yaw	1.05	0.17	1.93	0.032
Maxilla		X	-0.03	-0.38	0.32	0.819
Maxilla		Y	0.21	-0.42	0.84	0.371
Maxilla		Z	0.17	-0.08	0.42	0.116

Maxilla	Canines	roll	0.44	-0.21	1.10	0.122
Maxilla		pitch	-0.47	-2.47	1.52	0.504
Maxilla		Yaw	1.27	-0.47	3.01	0.103
Maxilla		X	0.04	-0.31	0.38	0.763
Maxilla		Y	0.23	-0.24	0.70	0.222
Maxilla		Z	0.02	-0.28	0.33	0.834
Maxilla	Premolars	roll	0.73	-0.85	2.30	0.239
Maxilla		pitch	-0.21	-1.71	1.29	0.685
Maxilla		Yaw	1.09	-0.24	2.41	0.080
Maxilla		X	0.20	-0.09	0.48	0.112
Maxilla		Y	0.16	-0.15	0.48	0.191
Maxilla		Z	0.03	-0.09	0.14	0.503
Maxilla	Molars	roll	0.11	-1.48	1.71	0.837
Maxilla		pitch	0.15	-1.04	1.33	0.719
Maxilla		Yaw	-0.24	-2.28	1.81	0.739
Maxilla		X	0.18	-0.27	0.63	0.295
Maxilla		Y	-0.15	-1.04	0.74	0.636
Maxilla		Z	0.01	-0.25	0.26	0.943

X left/right translation, Y anterior tot posterior translation, Z cranial/caudal ,CI confidence interval, Significance was set at $\alpha = 0.05$. Significant differences indicated in red.
